# Diagnostic Point-of-Care Ultrasound (POCUS) for Abdominal Pain: A Case of Tumefactive Sludge

**DOI:** 10.7759/cureus.34506

**Published:** 2023-02-01

**Authors:** Ana Santos e Silva, Mafalda Sequeira, Maria Inês Santos, Luciana Silva, José Mariz

**Affiliations:** 1 Internal Medicine, Unidade Local de Saúde do Litoral Alentejano, Santiago do Cacém, PRT; 2 Serviço de Medicina Interna, Hospital Garcia de Orta, Almada, PRT; 3 Internal Medicine, Hospital Distrital de Santarém, Santarém, PRT; 4 Serviço de Medicina Interna, Centro Hospitalar de Vila Nova de Gaia e Espinho, Vila Nova de Gaia, PRT; 5 Emergency, Hospital de Braga, Braga, PRT

**Keywords:** benign gallbladder diseases, ultrasound imaging, abdominal pain, tumefactive sludge, pocus (point of care ultrasound)

## Abstract

Biliary sludge is an extremely viscous sediment, consisting essentially of calcium bilirubinate granules and cholesterol crystals, which, due to its high viscosity, has poor and slow movement, leading to a mass-like configuration called tumefactive biliary sludge. Tumefactive sludge was first described with the advent of ultrasonography in the 1970s and is an uncommon intraluminal lesion of the gallbladder (GB).

The differential diagnoses for an echogenic mass in the GB lumen include GB carcinoma, tumefactive sludge, and gangrenous cholecystitis. Ultrasonography is the election method for the screening of GB diseases, with diagnostic accuracy exceeding 90%. The point-of-care ultrasound (POCUS) has shown a major improvement in the evaluation of hepatobiliary diseases. POCUS allows the detection of GB wall thickness, pericholestatic fluid, sonographic Murphy's sign, and dilatation of the common bile duct. The authors present a case of abdominal pain caused by the presence of tumefactive sludge in the GB, in which POCUS helped establish the diagnosis and therapeutic guidance.

## Introduction

Abdominal pain is one of the main causes leading people to the emergency department and has a variable clinical outcome [1]. Recently, point-of-care ultrasound (POCUS) has shown an increasing importance in the evaluation of abdominal pain, particularly in the hepatobiliary field [1]. Gallbladder (GB) imaging is challenging, given its variability in size, shape, and position. With POCUS, it is possible to detect GB wall thickening, pericholestatic fluid, sonographic Murphy's sign, and dilatation of the common bile duct [1,2].

Tumefactive sludge translates the precipitation of solid particles from bile and has a global estimated incidence of 2% with higher numbers often associated with pregnancy, patients with rapid weight loss, critically ill patients under parenteral nutrition, drugs such as Ceftriaxone and Octreotide and those with sickle disease or other causes of hemolysis [2,3]. Tumefactive sludge has a benign evolution, although it may be associated with complications such as biliary colic, acute cholangitis, and pancreatitis. Medical therapy is limited, and when patients are symptomatic or if complications arise, cholecystectomy is indicated [2].

## Case presentation

A 79-year-old female, partially dependent on daily life activities (55 points on the Barthel Index), and a personal history of heart failure with preserved ejection fraction and class II on the New York Hearth Association scale, hypertension, atrial fibrillation, diabetes, status post-ischemic stroke with secondary vascular epilepsy, chronic obstructive pulmonary disease, obesity, dyslipidemia, and chronic gastritis, presented to the emergency department with colicky acute abdominal pain in the right hypochondrium starting approximately 48h prior to admission. The pain was described as 8/10 in intensity, no aggravating factors were noted but the pain gets better with analgesics. The patient also mentioned having anorexia and asthenia, while denying fever, vomiting, or other gastrointestinal symptoms.

On physical examination, the patient was hemodynamically stable, with mild signs of dehydration, afebrile, with a bulbous and distended abdomen, with positive murphy sign, and without clinical signs of peritonitis.

Blood analyses showed leukocytosis (10.0 x 10^9^ /L) with neutrophilia (74.5%) and increased C-reactive protein (147.6 mg/L). There was also mild hypernatremia on the ionogram (Na^+^ 148 mmol/L; normal range: 135-145 mmol/L) with no other relevant changes. Liver function tests were unremarkable.

**Figure 1:**
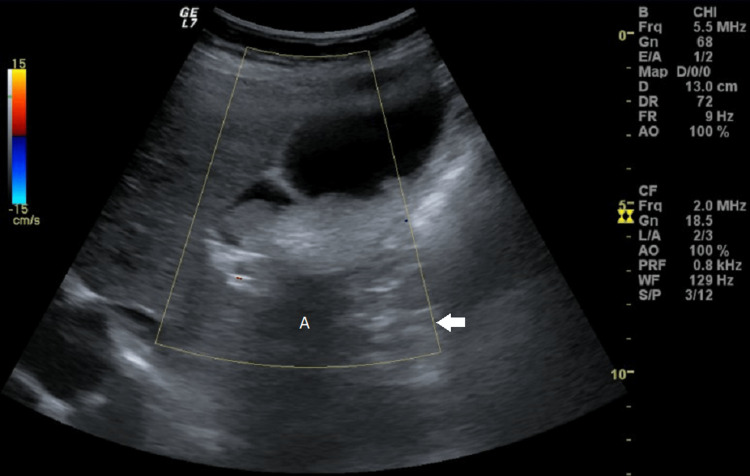
Ultrasound imaging of movable intraluminal echogenic mass-like gallbladder lesion, without posterior acoustic shadowing, internal vascularity absents by color Doppler ultrasound high attenuated. Arrow: Color Doppler field; A: Artifact resembling posterior acoustic shadowing but without typical features of gallstones

**Figure 2:**
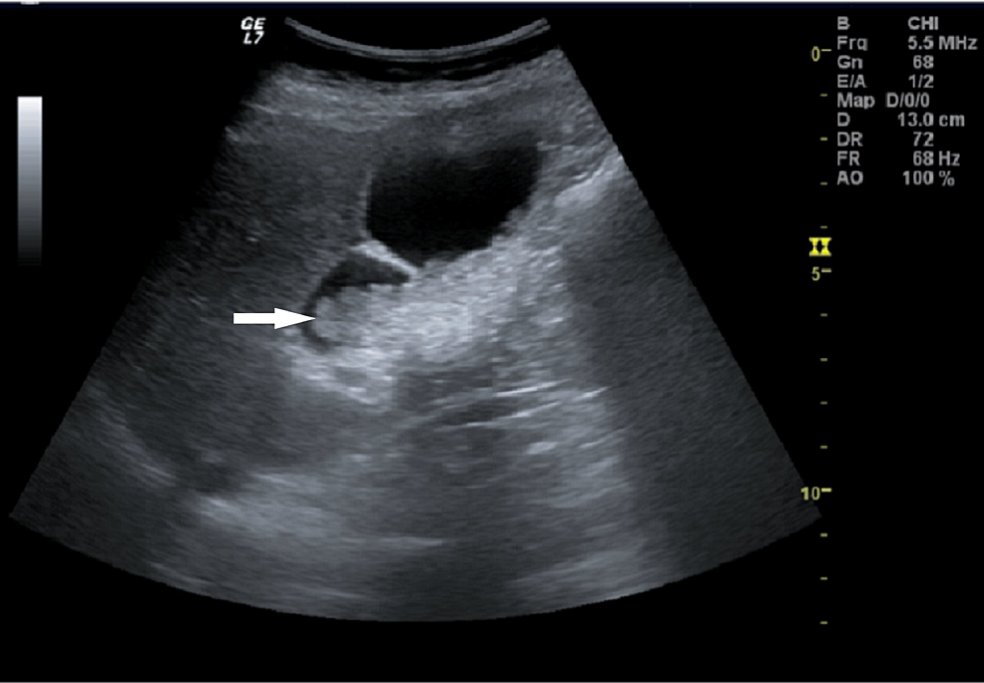
Longitudinal section of the gallbladder showing intraluminal echogenic mass-like Arrow: Gallbladder lesion

POCUS was promptly carried out by a dedicated POCUS team (consisting of an Internal Medicine assistant physician and Internal Medicine interns) in the ER and revealed a moderately distended GB, with regular unthickened walls, containing a low-level echoes polypoid mass in its dependent portion (round and lobulated) without acoustic shadowing (Figures 1, 2). Given these echographic signs, the hypothesis of biliary sludge was considered. The sonographic Murphy's sign was positive.

**Figure 3:**
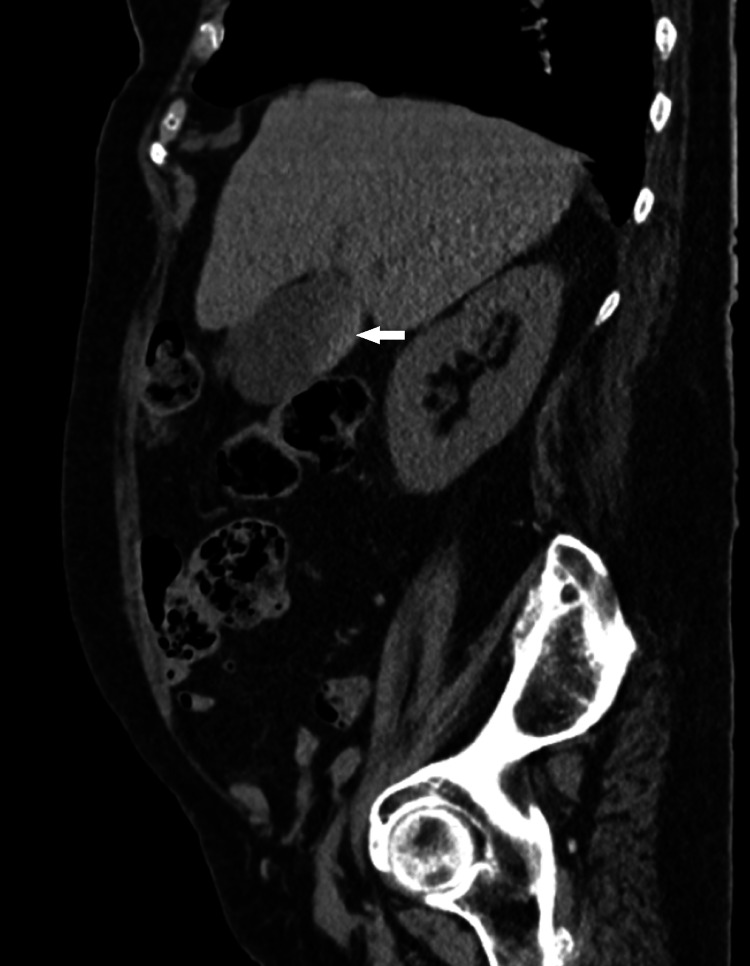
Intraluminal mass on pre-contrast thoraco-abdomino-pelvic computed tomography (TC scan) with no enhancement on post-contrast computed TC scan Arrow: Gallbladder lesion

The patient underwent thoraco-abdomino-pelvic computed tomography (CT scan) to better characterize the mass identified by POCUS and to exclude another potential infectious focus. The CT scan confirmed the presence of a mass‑like configuration with a pseudotumoral appearance, compatible with tumefactive biliary sludge (Figure 3). The patient was admitted to the Internal Medicine department and was treated with 12 days of antibiotic therapy with Piperacillin-Tazobactam due to cholecystitis secondary to biliary sludge. It was decided not to perform the immediate surgical intervention, given the clinical improvement of the patient with medical therapy. The patient was discharged, clinically improved, and awaiting a formal abdominal reassessment ultrasound.

## Discussion

GB and bile duct diseases are common in clinical practice and the most common pathology is gallstones [4]. Annually, about one million people are diagnosed with gallstone disease [4]. The formation of gallstones results from the precipitation of the main components of bile when they exceed their solubility concentration, and their main components are cholesterol and bilirubin [2-4]. Currently, the method of choice for evaluating GB disease is ultrasound, considering its safety, non-invasiveness, and real-time imaging, with a diagnostic accuracy of about 90% [1,3,5-7]. Despite this, it is important to note the subjectivity of the operator, the limitations associated with the body biotope, and the presence of intra-intestinal gas that may require the use of other imaging techniques, namely CT scan and MRI [2,4,6,7].

To improve decision-making capacity at the patient's bedside and optimize patient care, the use of POCUS in the emergency department has shown increasing importance, particularly in the hepatobiliary field [1,8]. Through POCUS, it is possible to assess the presence of GB wall thickening, pericholestatic fluid, common bile duct dilation, and the presence of perivesicular fluid, demonstrating a sensitivity of 89.8% and a specificity of 88.0% in the diagnosis of cholecystitis [1,8]. Despite the high sensitivity and specificity of ultrasound for GB diseases, differential diagnoses of less common pathologies depend on other imaging methods [3,4,7].

The biliary sludge is an extremely viscous sediment, consisting essentially of calcium bilirubinate granules and cholesterol crystals, which appear in ultrasound-like low-level echoes that lay in the dependent portion of the GB without acoustic shadowing [2,3,6]. Due to its high viscosity, the movement of the biliary sludge is poor or very slow, which often leads to a mass-like configuration, called tumefactive biliary sludge [3,7]. In view of this pseudotumor configuration associated with the absence of movement detected on ultrasound, a tumefactive biliary sludge is often confused with a polypoid vesicle or with neoplastic vesicular disease [3,6,7,9]. Despite its low sensitivity, the absence of a doppler signal at the level of the lesion helps to differentiate it from neoplastic pathology, typically characterized by high vascularization [5]. Although described as a rare pathology, with the increasing use of ultrasound in clinical practice, an increase in the prevalence of 0.18%-0.27% has been observed [5].

The clinical situations associated with a higher incidence of biliary sludge are pregnancy, rapid weight loss, hemolysis, prolonged fasting, especially in critically ill patients, and the use of total parenteral nutrition [2,3,5,10]. Recently, new studies have shown the association of biliary sludge in patients with dementia, with a special association with Alzheimer's disease [10]. This association seems to be related not only to changes in eating habits but also to dysphagia and gastrointestinal motility disorders [10]. The previous facts make the recognition of tumefactive sludge and its normal benign course more and more imperative, as well as the eventual emergence of pharmacological therapy that prevents possible complications [2,10]. With the advancement of imaging techniques, an association between tumefactive sludge and bile duct neoplasms has been demonstrated, with an unknown prevalence, but with a higher risk in patients with old age, female sex, and patients with absence of hyperechoic spots within the sludge [5]. In these patients, a more careful follow-up for an earlier diagnosis of neoplastic disease is essential [3]. Despite these associations, the true clinical significance of tumefactive sludge remains unknown [5]. Clinical evolution depends on its etiology and, in the majority of patients, a complete recovery is seen when the precipitant factor disappears [2].

Medical therapy is limited. However, the use of drugs namely ursodeoxycholic acid, cholecystokinin, and stimulation of the GB have shown some benefits [2,10]. In the presence of associated symptoms or documented complications, cholecystectomy is the definitive treatment [2].

## Conclusions

The echographic image of biliary sludge can be easily mistaken by a neoplastic pathology or a large calculus of the GB, making its recognition important considering its benign evolution and potential spontaneous resolution. When an echogenic mass in the GB is identified, tumefactive sludge must be considered. When these lesions are discovered, the examination and ultrasound must be repeated out of the acute phase.

The vesicular POCUS plays a key role in the evaluation of patients with right upper quadrant abdominal pain, given it can be decisive in the immediate therapeutic decision (medical versus surgical). The technical mastery of abdominal POCUS in the ER is essential to define priorities in acute situations.
